# Effects of first-line biologic immunomodulation compared with non-biologic therapies in adult-onset Still’s disease: a two-center cohort study

**DOI:** 10.1186/s13075-026-03861-3

**Published:** 2026-07-20

**Authors:** Ruru Guo, Xuesong Liu, Yixuan Li, Yi Qin, Xinyu Li, Georg Schett, Liyang Gu, Jürgen Rech, Liangjing Lu

**Affiliations:** 1 Department of Rheumatology, Renji Hospital, Shanghai Jiao Tong University School of Medicine, Shanghai, China; 2 Department of Ultrasound, Renji Hospital, School of Medicine, Shanghai Jiaotong University, Shanghai, China; 3 Department of Internal Medicine 3-Rheumatology and Immunology, Friedrich- Alexander-Universität Erlangen-Nürnberg and Universitätsklinikum Erlangen, Erlangen, Germany; 4 Deutsches Zentrum Immuntherapie, Friedrich-Alexander-Universität Erlangen- Nürnberg and Universitätsklinikum Erlangen, Erlangen, Germany; 5 Center for Rare Diseases Erlangen, Friedrich-Alexander-Universität Erlangen- Nürnberg and Universitätsklinikum Erlangen, Erlangen, Germany

**Keywords:** Adult-onset Still’s disease, First-line treatment, Biologics, Non-biologic immune modulators

## Abstract

**Background:**

To evaluate comparative effectiveness of methotrexate (MTX), calcineurin inhibitors (CNI), Janus kinase inhibitors (JAKi), and biologics for the first-line therapies in adult-onset Still’s disease (AOSD) in real-world settings.

**Methods:**

Two AOSD cohorts were retrospectively analysed. First, effectiveness of first-line biological versus non-biologic modulator was validated using overlap weighting of propensity scores in the Shanghai AOSD cohort. To compare AOSD treatment strategies (MTX, CNI, JAKi, biologics), we pooled data from the Shanghai and Erlangen cohorts, emulated a target trial, and applied doubly robust weighted regression to adjust for demographic and clinical confounders. The primary outcome was sustained event-free remission over 12 and 72 weeks.

**Results:**

124 AOSD patients were analyzed, 96 from the Shanghai and 28 from Erlangen cohort. In overlap-weighted analyses of Shanghai cohort, biologic was associated with higher sustained event-free remission and event-free state than non-biologic immune modulators (*P* = 0.0065 and *P* = 0.0096). In the pooled analysis, biologics were linked to higher likelihood of event-free state and sustained event-free remission at weeks 12 and 72 (all *P* < 0.05). Pairwise comparisons confirmed the advantage of biologics over CNI (sustained event-free remission at week 72 OR 0.11, *p* = 0.001), with significant benefits over MTX (OR 0.12, *p* = 0.002) and JAKi (OR 0.14, *p* = 0.008) emerging at week 72 for sustained event-free remission, and more frequent glucocorticoid discontinuation than MTX and CNI (both *p* < 0.05).

**Conclusions:**

First-line biological treatment is associated with improved sustained event-free remission compared to non-biologic treatments, such as MTX, CNI and JAKi and associated with more favorable long-term outcomes in AOSD.

**Supplementary Information:**

The online version contains supplementary material available at https://doi.org/10.1186/s13075-026-03861-3.

## Background

Still’s disease (SD), including adult-onset Still’s disease (AOSD) and systemic juvenile idiopathic arthritis, is a rare systemic autoinflammatory disorder characterized by dysregulated innate immunity and excessive cytokine release, particularly IL-1, IL-6, IL-18 and interferon-γ [1]. In recent years, treatment strategies have shifted from glucocorticoids and conventional modulators toward targeted biologics, reflecting an evolving paradigm of precision therapy [2]. A retrospective propensity-weighted cohort reported that first-line biologic therapy was associated with improved disease control compared with conventional modulators in Still’s disease [3]. However, the conventional immune modulator group in that study was limited to a small number of patients (12 treated with MTX, 30 with glucocorticoid monotherapy, and one with intravenous immunoglobulin), which may not provide a comprehensive representation of the effectiveness of conventional immune modulators in routine practice. Nevertheless, due to the rarity and heterogeneity of Still’s disease, existing randomized and observational studies remain limited by small sample sizes and restricted generalizability. External validation in independent cohorts is therefore essential to define effective and context-appropriate strategies, especially in settings with variable drug accessibility.

Glucocorticoids are the traditional therapy in AOSD, inducing rapid response in about 60% of patients [4], but long-term use is limited by toxicity and the need for steroid-sparing approaches. MTX is the most commonly used conventional modulators in AOSD, showing efficacy in reducing disease activity and as glucocorticoid-sparing agent [4], leading to improvement of systemic and joint manifestations [5]. Calcineurin inhibitors (CNI), which disrupt NFAT-mediated cytokine transcription and suppress the IL-18 and interferon-γ pathway [6], have demonstrated benefits in severe AOSD and macrophage activation syndrome (MAS) [7], yet their use is restricted by toxicity. Janus kinase (JAK) inhibitors mitigate the effects of IL-6, IL-10, IFN-γ, IFN-α, and GM-CSF, strongly implicated in the AOSD pathogenesis [8]. Preliminary evidence suggests potential utility of JAKi in AOSD complicated by MAS [2, 9]. Biologics targeting IL-1, IL-6, IFN-γ, and IL-18 have reshaped treatment paradigms, showing rapid and sustained efficacy [1], though evidence remains largely limited to small studies. This evolving therapeutic landscape highlights the heterogeneity of AOSD, clinically and pathogenically.

An important limitation of these clinical studies is their low representativeness of the general AOSD population. Current EULAR-PReS recommendation for the use of biologics as a first-line therapy heavily rely upon efficacy data in children [10]. Another issue is the lack of head-to-head comparisons among these immunosuppressants in AOSD, related to its nature as rare disease. Consequently, guidelines that focused on studies in adults did not find enough evidence to recommend biologics over conventional immune modulators as a first-line therapy in AOSD irrespective of disease activity [3]. In fact, clinical subsets of AOSD have been identified, potentially differing in pathogenesis and therapeutic response [11, 12]. It remains to be established whether AOSD heterogeneity is associated with different responses to biological and conventional immune modulators.

Given these limitations and scarce data guiding treatment decisions in routine practice, we established a two-center real-world observational study to test whether biologic provide an advantage as first-line therapy of AOSD.

## Methods

### Study design and participants

We performed a two-center, retrospective, propensity-weighted cohort study as described by Kernder and colleagues [3]. Patients with AOSD were identified by chart review at Center A (Shanghai, China) and Center B (Erlangen, Germany), with a data extraction window of January 1, 2024 to July 31, 2025. Only patients whose index visit occurred sufficiently early within this window to allow at least 72 weeks of follow-up before data extraction were eligible; those who did not meet this criterion were excluded. Eligibility criteria mirrored those previously described [3]: patients with active AOSD fulfilling Yamaguchi criteria and complete longitudinal data were included, excluding those < 18 years, treated with etoposide/experimental agents, pregnant, or non-adherent. Among 330 patients screened, 206 were excluded (6 deaths due to MAS-related multiorgan failure or severe infection during initial hospitalization; 12 misdiagnoses; 26 with incomplete data; 51 lost to follow-up; and 111 with insufficient follow-up). The final analysis included 124 patients (96 Shanghai, 28 Erlangen). The study protocol, including prespecified endpoints and analysis plan, was finalized before data extraction. The institutional review board of Renji Hospital approved the protocol (approval number 2016083). The Erlangen data were previously published [3] and fully anonymized; this study performed secondary analyses, and no additional ethical approval was required. All data were de-identified to ensure privacy and confidentiality.

### Data collection and variables

Clinical data were recorded by trained rheumatologists at each site using a standardized case report form developed in Erlangen. We collected demographics, disease features, laboratory markers, treatments, and predefined outcomes at baseline (diagnosis or flare), week 12 (range 6–24), and week 72 (range 60–100). Potential confounders for propensity score modelling were prespecified (sex, age, Pouchot score, ferritin) [3]. Pouchot score (range, 0–12) for each patient was assessed according to the criteria described by Pouchot et al. [13]. Data quality and plausibility checks were conducted centrally, with discrepancies resolved by consensus.

### Outcomes

The primary endpoint was sustained, event-free remission between weeks 12 and 72, as defined by Kernder and colleagues [3]. Sustained remission required clinical and biochemical quiescence (C-reactive protein < 10.0 mg/L, no arthritis, rash, or fever), while events included major treatment- or disease-related complications, MAS, death, or treatment escalation, with MAS diagnoses confirmed per current recommendations. MAS was identified using 2016 classification criteria [14]. Secondary outcomes, comprised individual components of the primary endpoint, glucocorticoid use (discontinuation, dose reduction, and dosage at last follow-up), and a modified primary endpoint excluding treatment switches. Additional analyses incorporated pooled Erlangen data to allow overall and pairwise comparisons across MTX, CNI, JAKi, and biologics.

### Calculation of exposure to MTX, CNI, JAKi, and biological immune modulatorsCalculation of exposure to 

Exposure to MTX, CNI, JAKi, and biologics was defined by recorded treatment periods; discontinuation was assumed at the first visit without documentation. For patients with multiple episodes of the same drug class, only the earliest was analysed, and those sequentially exposed to different treatment classes were classified by the first eligible exposure. Patients receiving combinations of non-biologic modulators or switching between them at last follow-up were excluded. Concomitant glucocorticoid use was permitted in all groups, and MTX use with biologics was allowed, with such patients classified in the biologics group and MTX considered a confounder in weighted analyses.

### Statistical analysis

Baseline characteristics were described using means (SD) or medians (IQR) for continuous variables and counts (%) for categorical variables, with group comparisons via t tests, Wilcoxon rank-sum, χ², or Fisher’s exact tests as appropriate. To address confounding by indication, we applied propensity score-based weighting. For the comparison of first-line non-biologic vs. biologics, overlap weighting (OW) was used, as inverse probability weighting yielded residual imbalance (absolute standardized mean differences > 0.20). OW reweights the study population to emphasize the region where treatment groups have comparable baseline characteristics [15]. By down-weighting individuals with extreme propensity scores, those highly likely or unlikely to receive a given treatment, OW achieves covariate balance and enables valid comparisons across groups, even when the original sample sizes are unequal [16]. For the four-group comparison (MTX, CNI, JAKi, and biologics), generalized overlap weights were estimated from a multinomial propensity model. Covariate balance was assessed using standardized mean differences and graphical diagnostics.

Treatment effects were estimated within a doubly robust framework. To address baseline imbalances, OW was applied to generate a pseudo-population with balanced covariate distributions. Outcomes were analyzed with survey-weighted regression models (survey::svyglm), with therapy type as the independent variable and baseline covariates included. Binary outcomes were assessed using weighted logistic regression and continuous outcomes with weighted linear regression.

Sensitivity analyses were performed using Firth’s penalized logistic regression for sparse binary outcomes and robust MM-type regression (lmrob) for continuous outcomes, to account for potential small-sample bias, separation, and influence of outliers. Additional robustness checks included alternative weighting strategies and complete-case analyses. All analyses were performed in R (version 4.5.1) with standard packages for propensity weighting, survey regression, and sensitivity modelling.

## Results

Of the 330 patients screened, 124 met the eligibility criteria in total, comprising 96 from the Shanghai cohort and 28 from Erlangen cohort. The Shanghai cohort was used for validation, and the full cohort was analyzed for overall and pairwise comparisons across the four treatment regimens (Supplementary Fig. 1).

### Cohort characteristics

Of the 96 patients in the Shanghai cohort (validation cohort), 83 (86%)/96 patients were female and 13 (14%) were male. All patients were of Asian ethnicity. The mean age at inclusion was 40.8 years (SD 15.7), and the median duration of symptoms before diagnosis was 2.0 months (IQR 0.7-7.0) in Shanghai cohort. The most frequent clinical manifestations at diagnosis were fever (95 [99%] of 96 patients), arthralgia (81 [84%]), and rash (83 [86%]). Median CRP at diagnosis was 62.9 mg/L (IQR 27.3-109.3) and median ferritin concentration was 3982 µg/L (IQR 1581–8450). Pulmonary involvement was observed in 14 patients, most commonly as scattered pulmonary infiltrates with pleural effusion (9/14). The remainder comprised organising pneumonia with pleural thickening and effusion (2/14), interstitial pneumonia with pulmonary oedema and pulmonary hypertension (1/14), bilateral pulmonary infiltrates with focal fibrosis (1/14), and bilateral pulmonary infiltrates with left-ventricular localized fibrosis and pericardial effusion (1/14). Regarding treatment, 84 (87.5%) patients received first-line non-biologic immunomodulators and 12 (12.5%) received first-line biological immunomodulators (all tocilizumab). No patient underwent treatment switching, as anakinra was not available in China before 2025. All patients received glucocorticoids (median initial dose 90 mg/day, IQR 60-145.8 mg/day), and 7 (7.3%) additionally received intravenous immunoglobulin (IVIG) (Supplementary Table 1).

In the Erlangen cohort, 28 patients were included, comprising 11 male and 17 female, all of Caucasian ethnicity. The median duration of symptoms before diagnosis was 2.0 months (IQR 1.0–17.0). The most common clinical manifestation was fever (28/28 [100%]), followed by rash (26/28 [92.9%]). Median CRP concentration was 123.3 mg/L (IQR 76.1–190.0), and median ferritin concentration was 2228 µg/L (IQR 997.5–5718). No patients had pulmonary involvement. All patients received anakinra as first-line biological treatment, with 18 patients receiving concomitant corticosteroids (median initial dose 35 mg/day [IQR 0-60 mg/day]). No patients received intravenous immunoglobulin.

### Confounding covariates for multivariable analysis

Confounder selection for this external validation cohort was consistent with the German study and is shown in Supplementary Table 2. In our cohort, patients receiving non-biologic had a higher mean serum ferritin concentration (7296.7 µg/L [SD 10731.7]) compared with those receiving bDMARD (4154.7 µg/L [SD 2318.1]; Supplementary Table 2). Weighted regression models were applied, with treatment regimen as the independent variable and respective outcomes as the dependent variables. After overlap weighting, the effective sample size was 30 for the non-biologic group and 11 for the biologic group. For ease of interpretation, rounded values are reported.

### Effectiveness of non-biologic vs. biologic immune modulators after overlap weighting

**Fig. 1:**
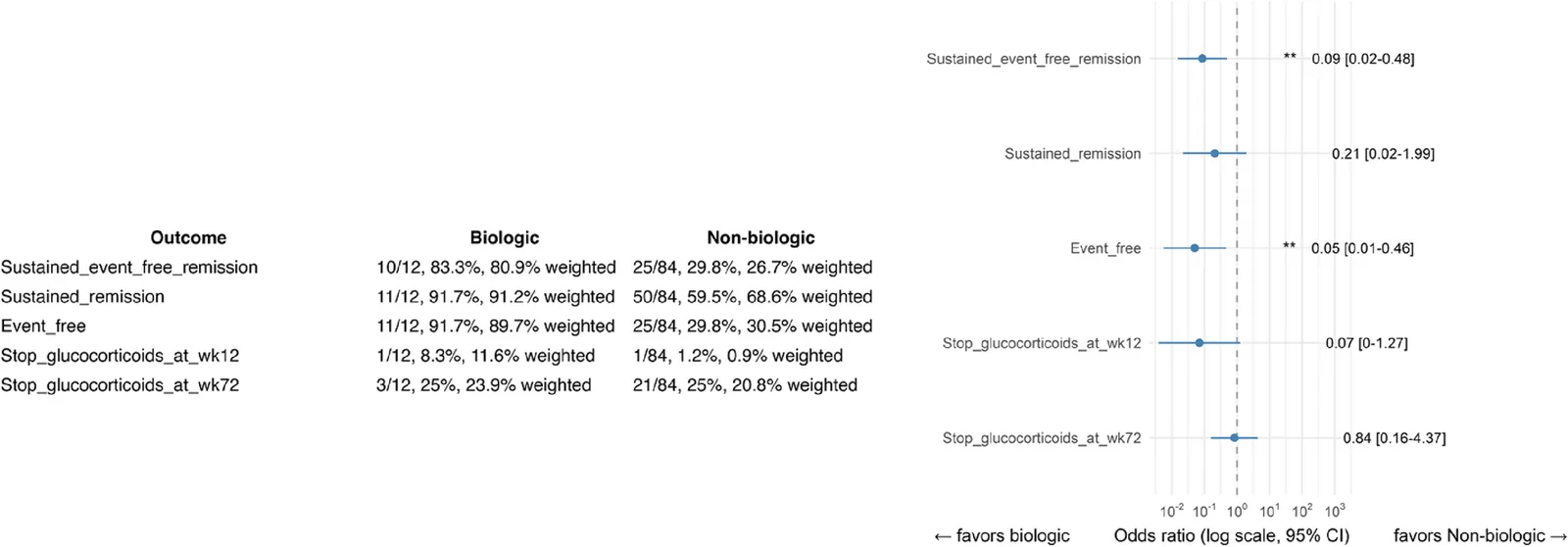
Odds ratios for the primary and key secondary endpoints in validation cohort. Odds ratios estimated by survey-weighted logistic regression analysis comparing first-line biological treatment with first-line non-biologic treatment in adult-onset Still’s disease patients (^**^p < 0.01). Non-biologic treatment was used as the reference group; odds ratios below 1 indicate superiority of biologic treatment

**Fig. 2:**
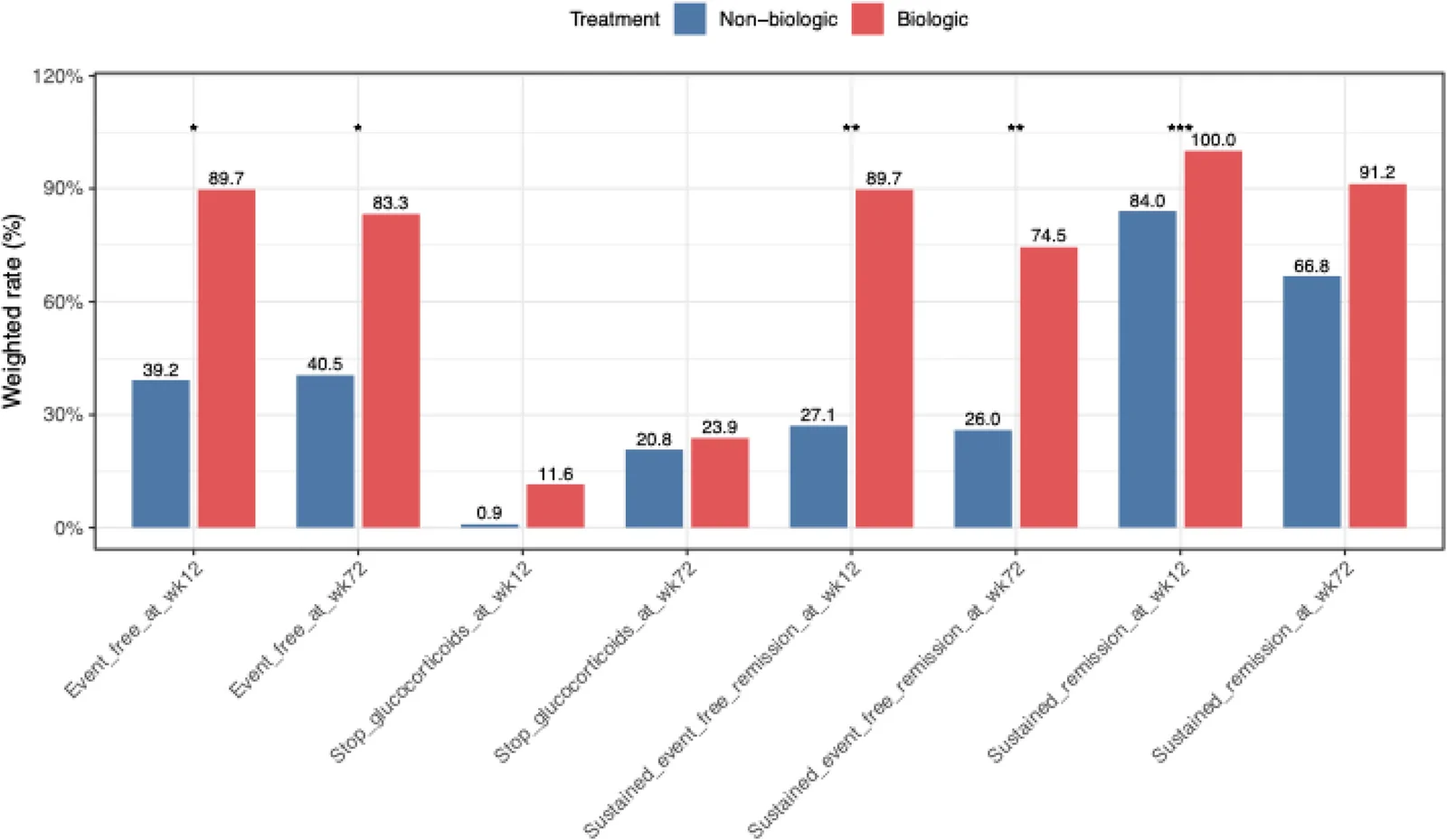
Proportion of patients reaching the key secondary endpoints. Weighted rates of outcomes at weeks 12 and week 72 in AOSD patients receiving first-line biological and first-line non-biologic immune modulator treatment; vertical axis shows weighted rates (%) and p values are from survey-weighted logistic regression (^*^p < 0.05, ^**^p < 0.01, and ^***^p < 0.001)

After overlap weighting, first-line treatment with biological immune modulators was associated with higher likelihood of achieving the primary endpoint of sustained, event-free remission (odds ratio [OR] 0.09, 95% CI 0.02–0.48; *p* = 0.0065; Fig. 1) as well as the secondary endpoint of event-free status (OR 0.05, 95% CI 0.01–0.46; *p* = 0.0096). For the remaining endpoints shown (sustained remission, glucocorticoid discontinuation at week 12 and at week 72), point estimates also favored biological immune modulators, but their 95% CIs included the null and therefore did not achieve statistical significance. The proportion of patients achieving sustained, event-free remission was higher in the first-line biological immune modulator group compared with patients receiving first-line non-biologic immune modulators at both week 12 (89.7% vs. 27.1%; *p* = 0.0059) and week 72 (74.5% vs. 26.0%; *p* = 0.0081). Similarly, the proportion of patients remaining event-free was greater in the first-line biological immune modulator group at week 12 (89.7% vs. 39.2%; *p* = 0.023) and week 72 (83.3% vs. 40.5%; *p* = 0.029), with all comparisons reaching statistical significance (Fig. 2). In a sensitivity analysis excluding JAKi from the non-biologic comparator arm (biologic, *n* = 12 vs. MTX and CNI, *n* = 61), the association remained advantage across primary and key secondary endpoints (Supplementary Table 3).

### Comparative baseline characteristics of treatment groups before and after overlap weighting

Overall, treatment with biological immune modulators was associated with higher rates of sustained remission and fewer adverse periods, prompting further pairwise comparisons among individual non-biologic treatments (MTX, CNI, JAKi) and biological treatments. We categorized patients into four treatment groups for comparison: MTX (*n* = 30, 24.2%), CNI (CsA or Tac, *n* = 31, 25%), JAKi (tofacitinib or baricitinib, *n* = 23, 18.5%) and biological treatments (anakinra or tocilizumab, *n* = 40, 32.3%). Subgroup analyses separating anakinra from tocilizumab were not performed due to small numbers. Baseline characteristics differed across the four treatment groups, reflecting treatment indications: the CNI group had the highest disease activity, ferritin, liver abnormalities, and glucocorticoid exposure; the JAKi group showed intermediate severity; the MTX group had the lowest disease activity and ferritin, with more females. Patients receiving biological treatments were predominantly male with highest CRP levels and neutrophil counts. Concomitant glucocorticoids were used in all patients with the exception of 10 patients receiving biological treatments. Eleven patients with biological treatments received a combination with MTX (Supplementary Table 4).

Selected baseline characteristics of patients across the four treatment groups are shown before and after propensity score weighting (Table 1). Before weighting, notable differences were observed in sex distribution, Pouchot score, symptom duration, serum ferritin, CRP, AST, neutrophil count, glucocorticoid dose and concomitant MTX or IVIG use. After weighting, these covariates were more balanced across groups, reflecting improved comparability for subsequent efficacy analyses. Weighted sample sizes increased slightly due to the reweighting procedure, with adjusted means or proportions reported for continuous and categorical variables, respectively.

**Table 1:** Selected baseline characteristics before and after overlap weighting

	Before weighting, *n* (%)				After Weighting, *n* (%)			
	MTX	CNI	JAKi	BIO	MTX	CNI	JAKi	BIO
N	30	31	23	40	45	62	56	78
Age, mean (SD)	40.5 (16.7)	41.0 (16.5)	39.2 (15.4)	40.7 (12.5)	42 (17.4)	38.2 (16.0)	39.4 (15.8)	42.6 (12.9)
Males, N (%)	2 (6.7)	4 (12.9)	2 (8.7)	16 (40.0)	3 (7.1)	11 (18.3)	4 (6.9)	26 (33.4)
Symptoms								
Rash N (%)	27 (90.0)	31 (100.0)	16 (69.6)	35 (87.5)	41 (89.5)	62 (100)	40 (71.6)	68 (87.6)
Pouchot score (0–12), mean (SD)	5.3 (1.3)	7.4 (1.8)	6.7 (2.1)	5.8 (2.0)	5.4 (1.4)	6.9 (1.9)	6.9 (2.1)	5.9 (2.0)
Symptom duration (months), mean (SD)	5.7 (12.2)	14.7 (45.2)	13.1 (18.3)	25.4 (51.7)	5.7 (12.3)	19.4 (50.8)	11.7 (17.7)	24.5 (50.7)
Laboratory parameters								
Ferritin (11–306 µg/L), mean (SD)	2645 (2410)	11,220 (13051)	8075 (11947)	8278 (21541)	2898 (2497)	9963 (12847)	8957 (12088)	9456 (25086)
CRP (0–5 mg/L), mean (SD)	85 (70)	67 (61)	52 (30)	128 (72)	95 (76)	72 (67)	56 (41)	118 (73)
AST (7–45 U/L), mean (SD)	89 (93)	213(254)	201(283)	64 (58)	90 (95)	184 (241)	216 (288)	68 (61)
Neutrophils (3.5–9.5 × 10⁹/L), mean (SD)	16.5 (6.9)	11.0 (6.0)	14.2 (7.7)	16.4 (6.6)	16.9 (7.0)	11.9 (6.1)	14.2 (7.7)	16.2 (6.5)
Medication								
Glucocorticoid dose (mg/day), mean (SD)	72 (31)	182 (137)	130 (98)	56 (57)	73 (31)	155 (127)	137 (103)	64(57)
Combined with MTX, N (%)	30 (100)	0 (0)	0 (0)	12 (30)	45 (100)	0 (0)	0 (0)	32 (41)
Immunoglobulins, N (%)	0 (0)	9 (29)	1 (4.4)	0 (0)	0 (0)	14 (23)	3 (5)	0 (0)

Baseline characteristics of patients in the methotrexate (MTX), calcineurin inhibitor (CNI), Janus Kinase inhibitor (JAKi) and biological (BIO) treatment groups are shown before and after propensity score weighting. Weighting balanced key covariates including age, sex, Pouchot score, symptom duration, laboratory parameters, and concomitant therapies, enabling valid between-group comparisons. AST, aspartate aminotransferase. N values in the After Weighting columns represent weighted pseudo-population sizes (sum of overlap weights) used for covariate balance assessment, not effective sample sizes

### Effectiveness of treatments with respect to primary and secondary endpoints

**Fig. 3:**
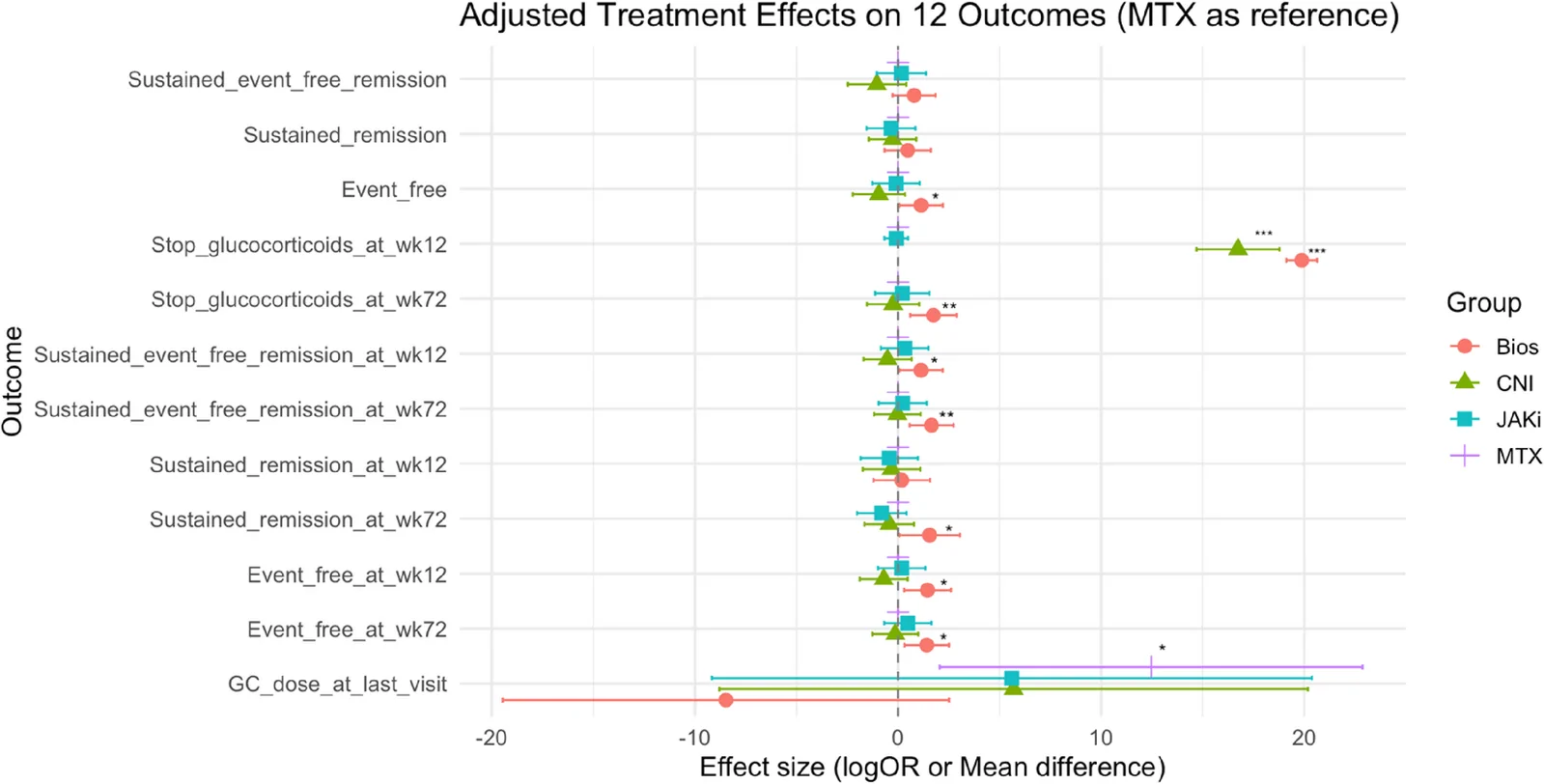
Multigroup comparison of the effectiveness of the various treatments. Forest plot showing the effect of treatment with calcineurin inhibitor (CNI), Janus Kinase inhibitor (JAKi) or biologicals (Bios) relative to methotrexate (MTX) treatment (reference) on outcomes in adult-onset Still’s disease. The vertical axis lists the outcomes, and the horizontal axis shows effect size (log odds ratio for binary outcomes or mean difference for continuous outcomes) with 95% confidence intervals. MTX was used as the reference group; effect sizes to the right of zero indicate superiority over MTX. Statistical significance is indicated by asterisks: ^*^p < 0.05, ^**^p < 0.01, ^***^p < 0.001

Baseline characteristics varied across the four treatment groups. Overall efficacy analyses were adjusted for expert-selected covariates (Pouchot score, age, sex, ferritin) and baseline variables with significant differences (rash, CRP, ALT, neutrophils, glucocorticoid dose, IVIG, concomitant MTX) using doubly robust weighted regression, with Firth logistic and robust linear models as sensitivity analyses. As shown in Fig. 3, biological treatments were associated with higher rates of event-free survival (OR 2.99, 95% CI 1.02–8.73, *p* = 0.048), sustained event-free remission at week 72 (OR 4.85, 95% CI 1.62–14.49, *p* = 0.006), sustained remission at week 72 (OR 4.69, 95% CI 1.07–20.56, *p* = 0.043), event-free status at week 12 (OR 4.18, 95% CI 1.32–13.28, *p* = 0.017), and event-free status at week 72 (OR 3.82, 95% CI 1.26–11.53, *p* = 0.019) compared to MTX. Biological treatments also significantly increased the likelihood of glucocorticoid discontinuation at weeks 12 and 72 (both *p* < 0.01). In contrast, patients on MTX had higher glucocorticoid doses at the last visit (β = 12.26, 95% CI 1.95–22.57, *p* = 0.022). At week 12, glucocorticoid discontinuation occurred in 15 patients in the biological treatment group, but only one patient in the CNI group, and no patient in the MTX or JAKi groups. This sparse distribution yielded inflated odds ratios with wide confidence intervals, indicating that the apparent difference should be interpreted with caution (Fig. 3).

### Pairwise comparisons of the effectiveness of MTX, CNI, JAKi, and biologics on primary and secondary endpoints

**Fig. 4:**
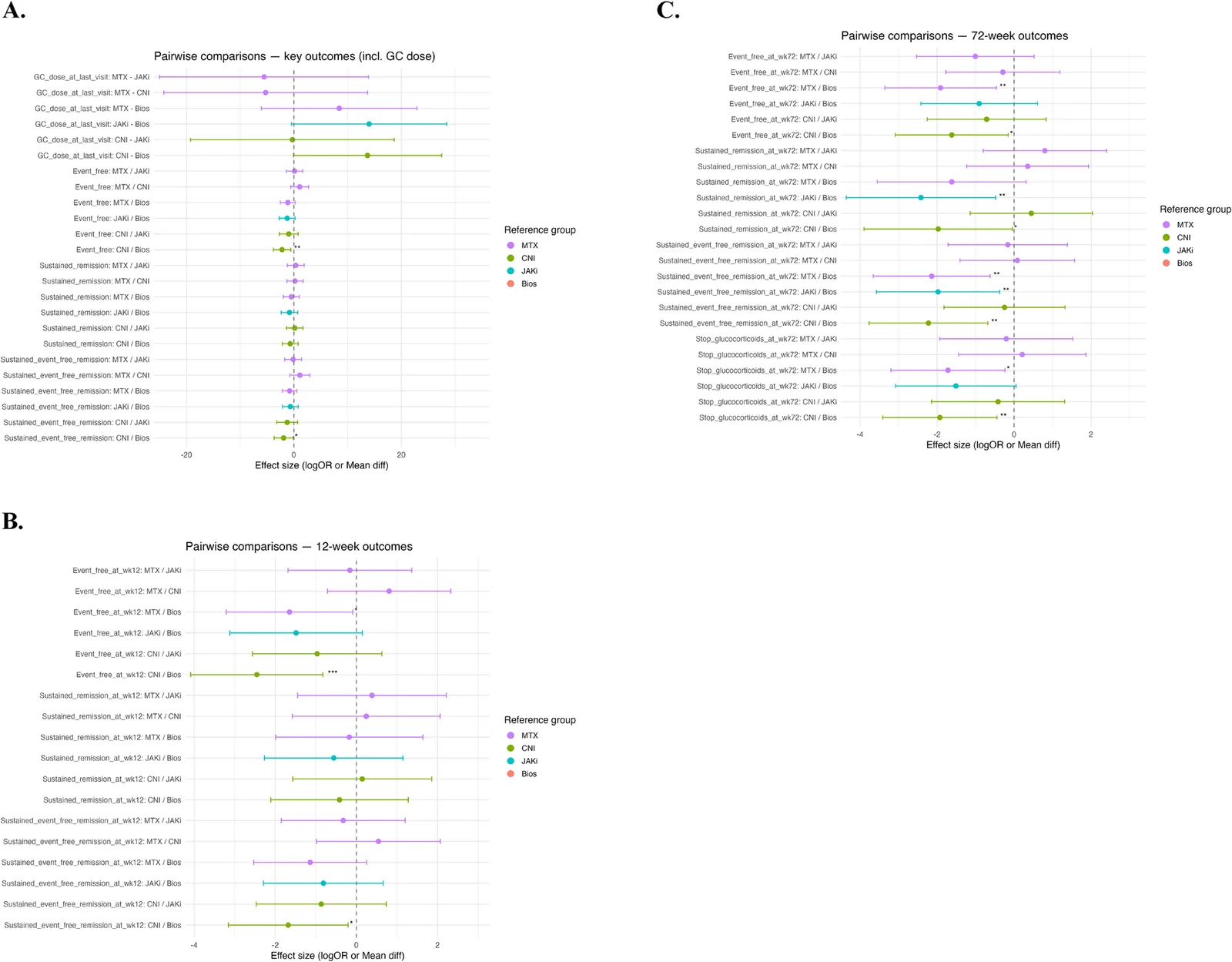
Pairwise comparisons of treatments on primary and secondary endpoints at overall, week 12 and week 72 follow-ups. **A** Primary outcome forest plot: pairwise comparisons of first-line treatment strategies for sustained event-free remission, sustained remission, and glucocorticoid discontinuation, showing weighted effect sizes (logOR or mean difference) and 95% CI. P values: *p<0.05, **p<0.01. **B** Secondary outcome forest plot at 12 weeks: pairwise comparisons of treatment groups for secondary endpoints at week 12; effect sizes with 95% CI. P values: *p<0.05, **p<0.01. **C** Secondary outcome forest plot at 72 weeks: pairwise comparisons for secondary endpoints at week 72; effect sizes (logOR or mean difference) with 95% CI. P values: *p<0.05, **p<0.01. Biologics were used as the reference group; effect sizes to the left of zero indicate superiority of biologic treatment over the comparator

Pairwise comparisons among the four treatment groups were performed using doubly robust weighted regression, with Firth logistic and robust linear models as sensitivity analyses for sparse samples. Biologics served as the reference group; odds ratios below 1 indicate lower rates in the comparator group. Over the entire follow-up (Fig. 4A), biologics were associated with significantly higher sustained event-free remission (CNI vs. biologics OR 0.15, 95% CI 0.02–0.86, *p* = 0.028) and event-free status (CNI vs. biologics OR 0.11, 95% CI 0.02–0.56, *p* = 0.003) than the CNI group. No statistically significant differences were observed in the remaining pairwise comparisons for these overall endpoints. At week 12 (Fig. 4B), biologics were associated with higher event-free status than both MTX (OR 0.19, 95% CI 0.04–0.91, *p* = 0.033) and CNI (OR 0.09, 95% CI 0.02–0.44, *p* = 0.001), and higher sustained event-free remission than CNI (OR 0.19, 95% CI 0.04–0.81, *p* = 0.018).

At week 72 (Fig. 4C), biologics showed the most consistent advantages. Sustained event-free remission was significantly higher in the biologic group than in all three comparators: MTX (OR 0.12, 95% CI 0.03–0.54, *p* = 0.002), CNI (OR 0.11, 95% CI 0.02–0.51, *p* = 0.001), and JAKi (OR 0.14, 95% CI 0.03–0.69, *p* = 0.008). Sustained remission was higher in the biologic group than in JAKi (OR 0.09, 95% CI 0.01–0.62, *p* = 0.007) and CNI (OR 0.14, 95% CI 0.02–0.96, *p* = 0.042). Event-free status was higher than in MTX (OR 0.15, 95% CI 0.03–0.63, *p* = 0.004) and CNI (OR 0.20, 95% CI 0.05–0.86, *p* = 0.024). Glucocorticoid discontinuation was more frequent in the biologic group than in MTX (OR 0.18, 95% CI 0.04–0.79, *p* = 0.015) and CNI (OR 0.15, 95% CI 0.03–0.64, *p* = 0.005).

## Discussion

By adopting the composite endpoints established in the German multicenter study [3], our work provides the first external validation in a large Chinese real-world cohort of AOSD patients. Using overlap weighting to adjust for prognostically relevant confounders and to approximate a randomized trial design, we found that biological immune modulators were associated with a higher likelihood of achieving the primary outcome of sustained event-free remission, with consistent advantages across several secondary endpoints compared with non-biologic immune modulators. Importantly, by jointly analyzing an international cohort (Erlangen and Shanghai patients), we were able to stratify non-biologic immune modulators into MTX, CNI, and JAKi, and for the first time conduct overall and pairwise comparisons with biological immune modulators. These analyses demonstrated that biological immune modulators were associated with better outcomes than CNI in terms of sustained event-free remission and event-free status, and were associated with higher sustained event-free remission rates than MTX, CNI, and JAKi in sustained event-free remission at later follow-ups, with a higher likelihood of glucocorticoid discontinuation compared to MTX and CNI treatments. Although the magnitude of benefit varied across outcomes, the overall direction of effect consistently favored biological immune modulators. Leveraging a doubly robust weighted regression framework (survey::svyglm) as the main analysis, with Firth logistic regression and robust estimators as sensitivity analyses, these findings extend beyond prior small series and meta-analyses, and highlight both the reproducibility and the granularity of treatment comparisons in AOSD across different populations and therapeutic contexts.

From a mechanistic perspective, AOSD is characterized by dysregulated activation of multiple pro-inflammatory cytokines, which provide the rationale for treatment with biological immune modulators [2, 17]. Evidence from small observational series and limited randomized trials, both in adult [18–21] and pediatric Still’s disease [22–24], consistently suggested the superiority of cytokine blockade, and meta-analyses have reported response rates of approximately 70–80% [3, 25]. In line with these findings, our study showed that 80.9% of patients treated with biological immune modulators achieved sustained event-free remission, thereby reinforcing the central role of cytokine inhibition in disease control. However, it remains uncertain which clinical phenotypes or disease trajectories should preferentially prompt the use of IL-1, IL-6R or IL-18 inhibitors. Notably, recent data indicate that IL-1 blockade may not confer protection against MAS [26], underscoring the heterogeneity of pathogenic pathways in AOSD. Given the diversity of treatment responses and the observation that most patients eventually achieve remission, it is plausible that distinct molecular endotypes drive disease progression [1, 12]. Defining these endotypes and their dominant cytokine signatures will be critical to inform more precise selection of biological therapies. This perspective may also partly explain the differential glucocorticoid-sparing effects observed across biologic agents. As suggested by Nigrovic and colleagues [27] in systemic JIA, early-phase IL-1β-dominant disease may respond more rapidly to anakinra with striking steroid-sparing, whereas patients with a hyperinflammatory endotype, as identified in our prior work [12], may follow different glucocorticoid discontinuation trajectories depending on their dominant cytokine signature.

Non-biologic immune modulators target diverse pathways reflecting AOSD’s mechanistic complexity; MTX primarily exerts anti-inflammatory effects via adenosine-mediated inhibition of pro-inflammatory mediators and is mainly used for chronic articular involvement and steroid-sparing [4]. Calcineurin inhibitors (cyclosporine, tacrolimus) inhibit NFAT-mediated T-cell activation, disrupt cytokine transcription, and are typically used for severe systemic AOSD or life-threatening complications [4, 7]. JAKi interfere with STAT activation downstream of multiple cytokines, offering a broader cytokine-blocking approach that has been used in refractory systemic and articular phenotypes [9, 28]. Clinical evidence for each class in AOSD remains limited to case series and small trials, with heterogeneous remission rates reported [1]. In our pairwise analyses the four treatment groups displayed distinct baseline phenotypes: MTX-treated patients were more often female with lower ferritin; CNI-treated patients had the highest Pouchot scores, more liver abnormalities and greater glucocorticoid exposure (consistent with more severe disease); JAKi-treated patients occupied an intermediate severity profile; and patients receiving biological treatments presented with markedly elevated inflammatory markers, further supporting the existence of clinically meaningful sub-phenotypes. These patterns suggest that clinicians may already be selecting agents according to presenting phenotype (articular versus high systemic inflammation), which could partly explain differential observed outcomes. In particular, CNI were preferentially used in the clinically severe patients, and the apparent advantage of biological treatments over CNI should therefore be interpreted with caution. These baseline imbalances are consistent with confounding by indication, which we addressed through overlap weighting and doubly robust regression to balance key prognostic covariates across groups (Table 1). Nonetheless, as the Pouchot score may not fully capture overall disease severity, residual confounding in the biologics-versus-CNI comparison cannot be entirely excluded. Importantly, after overlap-weighting and doubly-robust adjustment the association favouring biological immune modulators persisted; however, the optimal timing and context for choosing non-biologic versus biological immune modulators, so as to avoid empirical trial-and-error, remains unresolved and requires prospective validation.

Despite the study’s strengths, several limitations merit caution. Its retrospective design in a rare, heterogeneous disease constrained patient classification and covariate assessment, with small subgroup sizes reducing precision. Although overlap weighting and doubly robust models mitigated measured confounding, limited events and imbalanced group sizes produced wide confidence intervals, raising concerns about model stability and residual bias. Notably, glucocorticoid dose was adjusted for in the regression models but not included in the propensity score, so residual confounding from differential glucocorticoid exposure cannot be excluded. Group imbalances also restricted exploration of heterogeneity within treatment classes, and in the absence of randomized head-to-head comparisons, null pairwise findings should not be interpreted as evidence of no effect. Treatment assignment was structurally collinear with center and ethnicity, although a center-adjusted sensitivity analysis yielded consistent results (Supplementary Table 5). Inter-center variability, unmeasured confounders, and retrospective safety data further limit causal inference and generalizability. Finally, the disproportionate female representation (86%) in the Shanghai cohort likely reflects selection bias, as 70 (63%) of the 111 patients excluded for insufficient follow-up were male; this high exclusion rate most plausibly reflects a structural time constraint rather than outcome-related dropout, given the recent availability of biological agents for AOSD in China at the time of data extraction. These considerations highlight the need for larger pooled registries, prospective multicenter trials, and biomarker-driven studies to refine effect estimates, optimize treatment sequencing, and support personalized therapy in AOSD.

## Conclusions

In conclusion, this two-center study involving both Chinese and German cohorts provides observational evidence of an association between first-line biological therapy and improved outcomes in AOSD. Our findings suggest that biologics, when used as a first-line treatment, were associated with higher rates of sustained event-free remission compared with non-biologic immune modulators (MTX, CNI) and JAKi. Notably, the potential long-term benefit of biologics may extend beyond symptomatic control, as reflected by a higher rate of successful glucocorticoid discontinuation at 72 weeks. These results suggest that a “top-down” approach using biologics may improve clinical outcomes and reduce the long-term burden of steroid use in AOSD, though prospective validation is warranted given the observational nature of this study.

## Supplementary Information

Supplementary Material 1.

## Data Availability

No datasets were generated or analysed during the current study.
