# Supplementary material for: Effects of first-line biologic immunomodulation compared with non-biologic therapies in adult-onset Still’s disease: a two-center cohort study
Source: Arthritis Res Ther. 2026 Jul 20;28:181. doi: 10.1186/s13075-026-03861-3 (PMC13670786; doi:10.1186/s13075-026-03861-3)
Supplement: Supplementary file 1 [file 13075_2026_3861_MOESM1_ESM.docx]

**Supplementary Figure1: Patient selection flowchart**

**Supplementary Table1. Baseline characteristics of the Shanghai validation cohort**

|  | **Non-biologic Treatments (n=84)** | **Biologic Treatments**  **(n=12)** |
| --- | --- | --- |
| Age (years), mean (SD) | 40·0 (16·0) | 46·4 (12·4) |
| Sex |  |  |
| Male, N (%) | 8 (10) | 5 (42) |
| Female, N (%) | 76 (90) | 7 (58) |
| Ethnicity (Asian), N (%) | 84 (100) | 12 (100) |
| Symptoms | | |
| Fever*, N (%) | 84 (100) | 11 (92) |
| Arthralgia, N (%) | 70 (83) | 11 (92) |
| Arthritis, N (%) | 41 (49) | 7 (58) |
| Hepato/Splenomegaly, N (%) | 31 (37) | 3 (25) |
| Rash, N (%) | 74 (88) | 9 (75) |
| Leukocytosis^†^, N (%) | 71 (85) | 12 (100) |
| Pharyngodynia, N (%) | 66 (79) | 9 (75) |
| Lymphadenopathy, N (%) | 61 (73) | 8 (67) |
| Pouchot score^‡^, median (IQR) | 6.0 (5.0-8.0) | 7.5 (4.0-8.0) |
| Symptom duration (months), median (IQR) | 2.0 (0.7-6.8) | 7.0 (1.0-96.0) |
| Laboratory parameters | | |
| Ferritin (μg/L) ^§^, median (IQR) | 3984 (1548-8840) | 3856 (2447-5950) |
| CRP (mg/L), median (IQR) | 54 (24-103) | 106 (76-157) |
| CRP＞10mg/L^¶^, N (%) | 75 (89) | 12 (100) |
| AST (14-36 U/L), median (IQR) | 64 (40-178) | 35 (18-84) |
| ALT (7-45 U/L), median (IQR) | 48 (27-130) | 33 (18-52) |
| Neutrophils (3.5-9.5 × 10⁹/L), median (IQR) | 13.4 (8.9-17.3) | 17 (15-23) |
| Medication | | |
| Glucocorticoids, N (%) | 84 (100) | 12 (100) |
| Glucocorticoid dose (mg per day), median (IQR) | 90 (60-145) | 85 (65-150) |
| Methotrexate, N (%) | 30 (36) | 6 (50) |
| Ciclosporine/Tacrolimus, N (%) | 30 (36) | 0 (0) |
| JAK inhibitors, N (%) | 23 (27) | 0 (0) |
| Tocilizumab, N (%) | 0 (0) | 12 (100) |
| Immunoglobulins, N (%) | 6 (7) | 1 (8) |

Data are n (%), mean (SD), or median (IQR). CRP, C-reactive protein; *Fever greater than 39°C for at least 1 week. †Leukocytosis over 10 000/µL with more than 80% neutrophils. ‡Pouchot Score is a sum of AOSD manifestations ranging from 0 (no disease activity) to 12 (high disease activity). §Normal range 11–306 μg/L, markedly raised concentrations indicate active AOSD. ¶Normal range less than 5 mg/L, mild elevation 5–10 mg/L, moderate to marked elevation greater than 10 mg/L.

**Supplementary Table 2: Unweighted and weighted confounders**

|  | Over all (n=96) | First-line  non-biologic  treatment  therapy (n=84) | First-line biological  treatment  (n=12) | First-line  non-biologic  treatment  weighted  (n=30) | First-line biological  treatment  weighted (n=11) |
| --- | --- | --- | --- | --- | --- |
| Pouchot Score^*^, mean (SD) | 6.4 (2.0) | 6.5 (1.9) | 6.3 (2.4) | 6.2 (1.7) | 6.2 (2.4) |
| Male sex, N (%) | 13 (14) | 8 (10) | 5 (42) | 10 (34) | 4 (34) |
| Age (years), mean (SD) | 40.8 (15.7) | 40.0 (16.0) | 46.4 (12.4) | 46.0 (18.2) | 46.0 (12.9) |
| Ferritin (ug/L), mean (SD) | 6905 (10116) | 7296 (10731) | 4164 (2318) | 4147 (3429) | 4147 (2265) |
| Neutrophils (×10⁹/L), mean (SD) | 14.5 (7.1) | 13.8 (7.1) | 19.0 (5.5) | 18.4 (7.3) | 18.4 (4.8) |

*Pouchot Score is a sum of AOSD manifestations ranging from 0 (no disease activity) to 12 (high disease activity).

.

## **Supplementary Table 3. Sensitivity Analysis: Biologics vs Non-biologic (MTX+CNI only, JAKi excluded)**

Shanghai cohort only (Center=0). OW = overlap-weighted logistic regression; Firth = Firth penalized logistic regression (sensitivity for sparse outcomes).

| Outcome | OR_OW | CI_OW | P_OW | OR_Firth | CI_Firth | P_Firth | sig |
| --- | --- | --- | --- | --- | --- | --- | --- |
| Sustained_event_free_remission | 20.985 | 3.771-116.786 | 0.0009 | 12.600 | 3.206-71.047 | 0.0002 | *** |
| Sustained_remission | 8.021 | 0.883-72.893 | 0.0686 | 4.680 | 1.009-45.116 | 0.0485 | * |
| Event_free | 29.153 | 3.3-257.554 | 0.0034 | 14.442 | 3.114-139.585 | 0.0002 | ** |
| Stop_glucocorticoids_at_wk12 | 2.643 | 0.148-47.162 | 0.5107 | 5.261 | 0.401-69.307 | 0.1840 |  |
| Stop_glucocorticoids_at_wk72 | 1.160 | 0.251-5.359 | 0.8493 | 1.105 | 0.25-4.033 | 0.8854 |  |
| Sustained_event_free_remission_at_wk12 | 23.919 | 2.702-211.744 | 0.0057 | 13.459 | 2.907-129.957 | 0.0003 | ** |
| Sustained_event_free_remission_at_wk72 | 22.434 | 2.542-198.016 | 0.0066 | 12.560 | 2.717-121.17 | 0.0005 | ** |
| Sustained_remission_at_wk72 | 6.358 | 0.683-59.193 | 0.1086 | 3.261 | 0.694-31.599 | 0.1461 |  |
| Event_free_at_wk12 | 20.439 | 2.319-180.158 | 0.0083 | 9.618 | 2.088-92.578 | 0.0022 | ** |
| Event_free_at_wk72 | 9.849 | 1.825-53.154 | 0.0097 | 6.012 | 1.569-33.222 | 0.0076 | ** |

**Supplementary Table 4. Baseline characteristics of the treatment groups**

|  | **MTX** | **CNI** | **JAKi** | **BIO** | **p value** |
| --- | --- | --- | --- | --- | --- |
| N | **30** | **31** | **23** | **40** |  |
| Age (years), mean (SD) | 40.5 (16.7) | 41.0 (16.5) | 39.2 (15.4) | 40.7 (12.5) | 0.98 |
| Sex |  |  |  |  |  |
| Male, N (%) | 2 (6.7) | 4 (12.9) | 2 (8.7) | 16 (40.0) | 0.0016 |
| Female, N (%) | 28(99.3) | 27 (87.1) | 21 (91.3) | 24 (60.0) |  |
| Ethnicity |  |  |  |  |  |
| Ascian, N (%) | 30 | 31 | 23 | 12 |  |
| Caucasian, N (%) | 0 | 0 | 0 | 28 |  |
| Symptoms | | | | | |
| Fever, N (%) | 30(100.0) | 30(96.8) | 23(100.0) | 38(95.0) | 0.70 |
| Arthralgia, N (%) | 25 (83.3) | 25 (80.7) | 20 (87.0) | 37 (92.5) | 0.48 |
| Arthritis, N (%) | 9(30.0) | 19(61.3) | 13(56.5) | 20 (50.0) | 0.081 |
| Hepato/Splenomegaly, N (%) | 12 (40.0) | 14 (45.2) | 7 (30.4) | 15 (62.5) | 0.74 |
| Rash, N (%) | 27 (90.0) | 31 (100.0) | 16 (69.6) | 35 (87.5) | 0.0059 |
| Leukocytosis, N (%) | 26 (86.7) | 28 (90.3) | 18 (78.3) | 28 (70.0) | 0.14 |
| Pharyngodynia, N (%) | 23(76.7) | 24 (77.4) | 19 (82.6) | 30 (75.0) | 0.93 |
| Lymphadenopathy, N (%) | 20 (66.7) | 24 (77.4) | 17 (73.9) | 23 (57.5) | 0.31 |
| Pouchot score (0-12), mean (SD) | 5.3 (1.3) | 7.4 (1.8) | 6.7 (2.1) | 5.8 (2.0) | < 0.0001 |
| Duration (months), median (IQR) | 1.5 (0.7-5.3) | 1.0 (1.0-4.0) | 5.0 (1.0-12.0) | 2.5 (1.0-20 | 0.064 |
| Laboratory parameters | | | | | |
| Ferritin (11–306 μg/L) median (IQR) | 1686  (787.9-3513) | 8322  (6323-11728) | 3844  (1641-9586) | 3035  (1187-6089) | < 0.0001 |
| CRP (0-5 mg/L), median (IQR) | 71.9 (27.5-114.4) | 46 (21-108.2) | 43 (23.1-75.3) | 109.1 (74.8-184.0) | < 0.0001 |
| AST (14-36 U/L), median (IQR) | 51 (35-104) | 107 (56-295) | 59 (31-253) | 44 (28-79) | 0.0043 |
| ALT (7-45 U/L), median (IQR) | 43 (22-88) | 84 (32-211) | 50.0 (26-139) | 38 (21-54) | 0.022 |
| Neutrophils (3.5-9.5 × 10⁹/L), mean (SD) | 16.49 (6.9) | 10.98 (6.0) | 14.15 (7.7) | 16.43 (6.6) | 0.0036 |
| Medication | | | | | |
| Glucocorticoids, N (%) | 30 (100%) | 31 (100%) | 23 (100%) | 30 (75%) | <0.0001 |
| Glucocorticoid dose (mg per day), median (IQR) | 62 (40-97) | 133.0 (60-240) | 120 (80-150) | 50.0 (1-80) | <0.0001 |
| Methotrexate, N (%) | 30 (100) | 0 | 0 | 11 | <0.0001 |
| Ciclosporine/Tacrolimus,  N (%) | 0 | 31（100） | 0 | 0 |  |
| JAK inhibitors, N (%) | 0 (0) | 0 (0) | 23 (100) | 0 |  |
| Tocilizumab/ Anakinra, N (%) | 0 (0) | 0 (0) | 0 (0) | 40 (100) |  |
| Immunoglobulins, N (%) | 0 (0) | 9 (29.0) | 1 (4.4) | 0 (0) | <0.0001 |

Baseline characteristics of patients in the methotrexate (MTX), calcineurin inhibitor (CNI), Janus Kinase inhibitor (JAKi) and biological (BIO) treatment groups are shown. Data are n (%), mean (SD), or median (IQR). CRP, C-reactive protein; AST, Aspartate

Aminotransferase; ALT, Alanine aminotransferase.

## **Supplementary Table 5. Centre-Adjusted Sensitivity Analysis (MTX as reference)**

All models adjusted for centre (Shanghai vs Erlangen) in addition to IPTW weighting.

| Outcome | Group | Label | P |
| --- | --- | --- | --- |
| Sustained_event_free_remission | CNI | OR: 0.4103 (0.0906 - 1.858) | 0.2500 |
| Sustained_event_free_remission | JAKi | OR: 1.1344 (0.335 - 3.8419) | 0.8397 |
| Sustained_event_free_remission | Bios | OR: 11.6586 (2.0357 - 66.7693) | 0.0067** |
| Sustained_remission | CNI | OR: 0.7933 (0.2475 - 2.5434) | 0.6976 |
| Sustained_remission | JAKi | OR: 0.668 (0.2011 - 2.2185) | 0.5113 |
| Sustained_remission | Bios | OR: 8.6836 (0.9407 - 80.1601) | 0.0591 |
| Event_free | CNI | OR: 0.3959 (0.108 - 1.4508) | 0.1646 |
| Event_free | JAKi | OR: 0.8728 (0.2704 - 2.8171) | 0.8204 |
| Event_free | Bios | OR: 12.1486 (1.3368 - 110.4037) | 0.0285* |
| Stop_glucocorticoids_at_wk72 | CNI | OR: 0.7132 (0.1974 - 2.5764) | 0.6070 |
| Stop_glucocorticoids_at_wk72 | JAKi | OR: 1.2173 (0.3176 - 4.6659) | 0.7748 |
| Stop_glucocorticoids_at_wk72 | Bios | OR: 0.8035 (0.1578 - 4.0924) | 0.7927 |
| Sustained_event_free_remission_at_wk12 | CNI | OR: 0.6545 (0.1931 - 2.2178) | 0.4973 |
| Sustained_event_free_remission_at_wk12 | JAKi | OR: 1.3043 (0.4024 - 4.228) | 0.6587 |
| Sustained_event_free_remission_at_wk12 | Bios | OR: 18.2663 (2.004 - 166.4984) | 0.0112* |
| Sustained_event_free_remission_at_wk72 | CNI | OR: 0.9907 (0.3049 - 3.2194) | 0.9876 |
| Sustained_event_free_remission_at_wk72 | JAKi | OR: 1.1632 (0.3521 - 3.8426) | 0.8046 |
| Sustained_event_free_remission_at_wk72 | Bios | OR: 20.2734 (2.2159 - 185.485) | 0.0088** |
| Sustained_remission_at_wk12 | CNI | OR: 0.7795 (0.1938 - 3.1358) | 0.7264 |
| Sustained_remission_at_wk12 | JAKi | OR: 0.614 (0.1508 - 2.5002) | 0.4973 |
| Sustained_remission_at_wk72 | CNI | OR: 0.6805 (0.202 - 2.2924) | 0.5357 |
| Sustained_remission_at_wk72 | JAKi | OR: 0.4216 (0.1241 - 1.4318) | 0.1688 |
| Sustained_remission_at_wk72 | Bios | OR: 5.4583 (0.5842 - 50.9999) | 0.1393 |
| Event_free_at_wk12 | CNI | OR: 0.4962 (0.1512 - 1.6283) | 0.2500 |
| Event_free_at_wk12 | JAKi | OR: 1.1668 (0.3611 - 3.7705) | 0.7970 |
| Event_free_at_wk12 | Bios | OR: 9.9223 (1.0888 - 90.4227) | 0.0440* |
| Event_free_at_wk72 | CNI | OR: 1.4147 (0.4458 - 4.4894) | 0.5571 |
| Event_free_at_wk72 | JAKi | OR: 2.5799 (0.7948 - 8.3745) | 0.1173 |
| Event_free_at_wk72 | Bios | OR: 8.6845 (1.5312 - 49.2559) | 0.0161* |
| GC_dose_at_last_visit | CNI | Beta: 5.3978 (-8.6095 - 19.4051) | 0.4516 |
| GC_dose_at_last_visit | JAKi | Beta: 6.1107 (-8.6549 - 20.8763) | 0.4189 |
| GC_dose_at_last_visit | Bios | Beta: -7.6444 (-18.0997 - 2.811) | 0.1545 |
